# Long-term *Lactobacillus rhamnosus* BMX 54 application to restore a balanced vaginal ecosystem: a promising solution against HPV-infection

**DOI:** 10.1186/s12879-017-2938-z

**Published:** 2018-01-05

**Authors:** Ettore Palma, Nadia Recine, Lavinia Domenici, Margherita Giorgini, Alessandra Pierangeli, Pierluigi Benedetti Panici

**Affiliations:** 1 Department of Gynecological, Obstetric and Urologic Sciences, University “Sapienza” of Rome, Rome, Italy; 2 Department of Molecular Medicine, University “Sapienza” of Rome, Rome, Italy

**Keywords:** Probiotics, HPV infection, *Lactobacillus rhamnosus* BMX 54

## Abstract

**Background:**

Over recent years, a growing interest has developed in microbiota and in the concept of maintaining a special balance between *Lactobacillus* and other bacteria species in order to promote women’s well-being. The aim of our study was to confirm that vaginal *Lactobacilli* long-lasting implementation in women with HPV-infections and concomitant bacterial vaginosis or vaginitis might be able to help in solving the viral infection, by re-establishing the original eubiosis.

**Methods:**

A total of 117 women affected by bacterial vaginosis or vaginitis with concomitant HPV-infections were enrolled at Department of Gynecological Obstetrics and Urological Sciences, La Sapienza University, Rome, Italy between February 2015 and March 2016. Women were randomized in two groups, standard treatment (metronidazole 500 mg twice a day for 7 days or fluconazole 150 mg orally once a day for 2 consecutive days) plus short-term (3 months) vaginal *Lactobacillus* implementation (group 1, short probiotics treatment protocol group, *n* = 60) versus the same standard treatment plus long-lasting (6 months) vaginal *Lactobacillus rhamnosus* BMX 54 administration (group 2, treatment group, *n* = 57).

**Results:**

After a median follow up of 14 months (range 9–30 months) the chance to solve HPV-related cytological anomalies was twice higher in probiotic long-term users (group 2) versus short probiotics implementation group (group 1) (79.4% vs 37.5%, *p* = 0.041). Moreover, a total HPV-clearance was shown in 11.6% of short schedule probiotics implementation patients compared to a percentage of 31.2% in vaginal Lactobacilli long term users (*p* = 0.044), assessed as negative HPV-DNA test documented at the end of the study period.

**Conclusions:**

The consistent percentage of clearance of PAP-smear abnormalities and HPV-clearance obtained in long-term treatment group has been interestingly high and encouraging. Obviously, larger and randomized studies are warranted to confirm these encouraging results, but we believe that eubiosis re-establishment is the key to tackle effectively even HPV-infection**.**

**Trial registration:**

Retrospectively registered on PRS NCT03372395 (12/12/2017).

## Background

An increasing interest has been developed in microbiota, with the belief that probiotics could be able to promote women’s well-being and illnesses in several ways.

The vaginal microbiota of healthy women consists of a diversity of anaerobic and aerobic microorganisms (eubiosis). Lactobacilli are the most widespread and prevailing subpopulation. In some conditions, this balance can be compromised (dysbiosis) and other microorganisms may grow reducing anti-bacterial defence mechanisms. The loss of this delicate steadiness can move in numerous directions (pathobiosis), depending on different of factors (hormone levels, douching, sexual practices, bacterial interactions, host defences, and so on), promoting disorders such as bacterial vaginosis and yeast vaginitis, and then endorsing the occurrence of sexually transmitted diseases.

So, a steady vaginal ecosystem would have the ability to tackle infections, by maintaining a sort of local equilibrium between the different microbial subpopulations inhabiting vaginal micro-environment. Likewise, the microbial species that populate the vagina would play an important role in having Lactobacilli as the most remarkable protagonists of this process. The mechanisms by which Lactobacilli are able to stabilize the vaginal microbiota consist of the production of antimicrobial compounds (hydrogen peroxide, lactic acid, bacteriocin-like substances) and in the capability to adhere and compete for adhesion sites in the vagina with other pathogens [1–3].

Thus, we hypothesized that the development of any kind of disease/infection might be the result of a transitional process, beginning by compromising the physiological vaginal eubiosis, increasing Lactobacilli-mediated cytolysis and then reaching the stage of pathobiosis, when the vaginal ecosystem starts to be defenceless and becomes vulnerable to a variety of infections.

Although just putative relationships between dysbiosis/pathobiosis and cancer development/progression have been observed so far, the long duration of dysbiosis that precedes this condition and the hypothesis of possible combined effects with other risk factors, suggests the presence of greater clinical implications regarding the importance of microbiota conservation and balance [4–6]. Additionally, the association of microbial dysbiosis with several cancer types has been noted mainly in areas surrounded by mucosal membranes where bacteria live tightly [7, 8].

Infection with human papillomavirus (HPV) has been recognized as the major cause of cervical cancer development [9, 19]. Recently, a potential role of the vaginal dysbiosis/pathobiosis in promoting cervical HPV-related alterations and consequently precancerous lesions development through the elevation of pH has been reported [6, 8–10].

The aim of our study was to investigate if *Lactobacillus rhamnosus* BMX 54 (NORMOGIN ®) long-lasting vaginal application in women with dysbiosis and concomitant HPV-infections, might be able to have an advantageous effect on viral infection control, by restoring a stable microbiota to eubiosis.

To our knowledge this is the first trial in the literature to assess the efficacy of microbiota balance maintenance against HPV infections and related alterations.

## Methods

This is a pilot study, performed between February 2015 and March 2016 at Department of Gynecological Obstetrics and Urologic Sciences, “Sapienza” University of Rome. Inclusion criteria were: age > 18 years, documented BV or yeast vaginitis associated with HPV-infection documented as PAP-smear abnormalities (ASCUS, L-SIL or H-SIL histologically demonstrated as CIN1) and/or positive for HPV-DNA. Exclusion criteria were: pregnancy or breastfeeding, previous abnormal PAP-smear, CIN2–3, concomitant malignancies, immunological diseases, severe comorbidities, prolonged corticosteroid treatment.

Bacterioscopic exam, PAP-smear, HPV DNA test and colposcopy were performed for every enrolled patient at intake and every 3 months of follow up, when indicated. Bacterioscopic exam was performed every time by the same investigator as follows: each vaginal sample was rehydrated with normal saline solution and plotted under a phase contrast microscope with 400× magnification and an area of 0.016 mm^2^. The presence of bacteria, clue cells, number of vaginal leucocytes and other signs of possible microbial balance alteration/infections were recorded [11]. Evaluation of pH was made with Litmus paper test.

PAP-smears were evaluated by dedicated expert pathologists using the three-tiered CIN classification and Bethesda terminology [9]. An adequate smear was defined as a sample of adequate squamous cells with evidence of transformation zone noted on histologic examination.

HPV infection was detected using polymerase chain reaction (PCR) amplification of the viral DNA, followed by dot blot hybridization to identify its relevant subtypes including HPV 16, 18 and collectively other high-risk HPV subtypes (31–33–35-39-45-51-52-56-58-59-68).

Colposcopy assessment was performed on the basis of the terminology introduced by the Nomenclature Committee of International Federation for Cervical Pathology and Colposcopy in 2011 [12], identifying as *Grade 1* (minor changes) the occurrence of a fine mosaic, fine punctation, a thin acetowhite epithelium, or an irregular, geographic border and as *Grade 2* (major changes) the presence of a sharp border, an inner border sign, a ridge sign, a dense acetowhite epithelium, a coarse mosaic/punctation, or cuffed crypt openings. Atypical vessels or other suspicious signs of invasion (such as fragile vessels, irregular surface, exophytic lesion, necrosis, ulceration, tumor or gross neoplasm) were included under *Grade 2*. In the absence of abnormal findings, the colposcopic impression was designated as normal. The squamo-columnar junction visibility was classified as completely visible or non-completely visible. The transformation zone type was classified as type 1 and 2 (completely visible) or type 3 (not fully visible). Endocervical curettage was performed when the squamo-columnar junction was not completely visible. Lesion location was classified on the basis of histological results as follows: ectocervical (when the lesion was only present on ectocervical biopsy), endocervical (when the lesion was present on endocervical curettage), or ectocervical and endocervical (when the lesion was present on both).

**Fig. 1:**
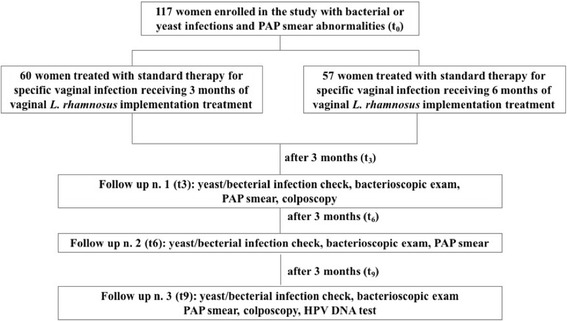
Flow chart. t_0_: time of inclusion; t_3_, t_6_ and t_9_: follow up time at 3, 6 and 9 months respectively

Patients were consecutively randomized in two groups, standard treatment plus short-term (3 months) vaginal Lactobacilli implementation (group 1, *n* = 60) vs standard treatment plus long-lasting (6 months) Lactobacilli administration (group 2, *n* = 57). Standard initial treatment for bacterial or yeast infections was metronidazole 500 mg (orally twice a day for 7 days) or fluconazole 150 mg (orally once a day for two consecutive days), respectively (Fig. 1).

We decided to administer probiotics as support to standard treatment in the short treatment protocol group as well, considering our encouraging results in vaginal infections control obtained in a previous study [13].

Lactobacilli were administered by vaginal tablets containing 10^4^ CFU/tablet freeze-dried *Lactobacillus rhamnosus* BMX 54, NORMOGIN ® as follows: once a day for 10 days, once every 3 days for 20 days and then once every 5 days for other 2 months in all patients (probiotics implementation for 3 months comprehensively). On the other hand, patients belonging to long-term treatment arm (group 2) continued using the same vaginal tablets once a week for a 5-month period, instead of just 2 months as others. All patients followed a strict follow-up (every 3 months for 9 months). HPV-DNA test was repeated at the end of the study period (Fig. 1).

The study was approved by the Ethical Committee and written informed consent was obtained from all patients.

Statistical analysis was performed using SPSS software package (SPSS Inc., Chicago, IL, USA) given as mean (SD) or number (%) of cases, with 95% confidence intervals (CIs), when suitable. Comparison of quantitative variables in the two groups considered was performed using the 2-sample Chi-square and Fisher’s test. The measures were indicated as mean ± SD and 95% confidence interval. All tests used were 2-tailed. A *p* value of <0.05 was considered as statistically significant.

## Results

A total of 117 women were included in the study (Fig. 1). Bacterial vaginosis was diagnosed following Amsel criteria [14]. Women were diagnosed with yeast vaginitis by the presence of vaginal discharge in association with at least one of the distinctive symptoms and signs of the disease (itching, burning, dyspareunia and dysuria) and by the presence of yeast infection by at least one of the following processes: wet mount preparation, Gram-staining or culture. Only patients with recognized yeast or bacterial infection and concomitant PAP smear alteration or HPV found by PCR analysis were enrolled in the study. Most of the patients had simultaneous bacterial vaginosis (55 and 54.4% in Group 1 and 2 respectively). Pap smear abnormalities were associated with HPV DNA positivity in 58.3 and 57.8% of cases in Group 1 and 2 respectively. Patients were consecutively randomized in two group as previously explained (Group 1, *n* = 60 and Group 2, *n* = 57).

Mean age in Group 1 was 32.4 years (10.5 SD, 95% CI 29.7–35.1), while in Group 2 it was 29.1 years (8.9 SD, 95% CI 26.7–31.5). Patients’ baseline characteristics are shown in Table 1. No significant differences were seen for any of them between Groups 1 and 2. A diagram of follow up schedule was shown in Fig. 1.

**Table 1:** Patients’ baseline characteristics

VARIABLES	GROUP 1 (*n* = 60)	GROUP 2 (*n* = 57)	*p*-value
Age, mean ± SD	32.4 ± 10.5	29.1 ± 8.9	NS
Use of contraceptive methods	35 (58.3%)	33 (57.8%)	NS
Regular menses	47 (73.3%)	48 (84.2%)	NS
Symptoms prevalence (vaginal itching and burning, dyspareunia and dysuria)	53 (88.3%)	49 (85.9%)	NS
Smoking	25 (41.6%)	19 (33.3%)	NS
Multiple partners	5 (8.3%)	6 (10.5%)	NS
Previous deliveries	31 (51.6%)	33 (57.9%)	NS
Bacterial vaginosis	33 (55.0%)	31 (54.4%)	NS
Yeast vaginitis	27 (45.0%)	26 (45.6%)	NS
Pap smear alterations	40 (66.6%)	39 (68.4%)	NS
Associated HPV DNA+	35 (58.3%)	33 (57.8%)	
HPV DNA+ alone	20 (33.3%)	18 (31.6%)	NS
HPV DNA+ patients (total)	55 (91.7%)	51 (89.4%)	NS
HPV DNA negative patients with PAP smear abnormalities	5 (8.3%)	6 (10.5%)	NS

*NS* not significant

Of all participants 84% (*n* = 98) reported being in a monogamous heterosexual relationship and 11 women reported same-sex relationships and eight declared not having had intercourses at the moment of interview. Compliance was excellent. All participants came back for all follow up visits.

Median follow up time was 14 months (range 9–30 months). After three months, there were no statistically significant differences between the two groups: remission rates of symptoms and bacterial or yeast infections related clinical findings were similar in the two cohorts (Group 1 93.3%, *n* = 56 and Group 2 96.4%, *n* = 55 respectively).

**Fig. 2:**
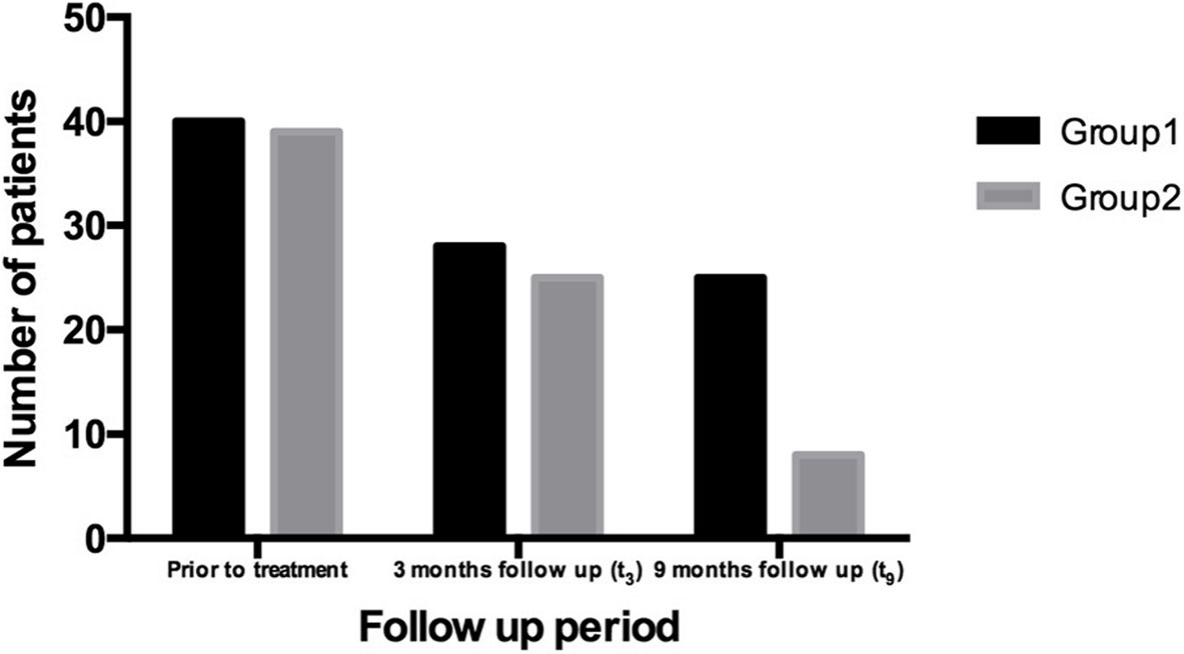
Trend of HPV abnormalities resolution in the two groups (group 1 – short probiotics implementation group, *n* = 60; group 2 – long probiotics implementation group, *n* = 57; *p* = 0.041)

**Fig. 3:**
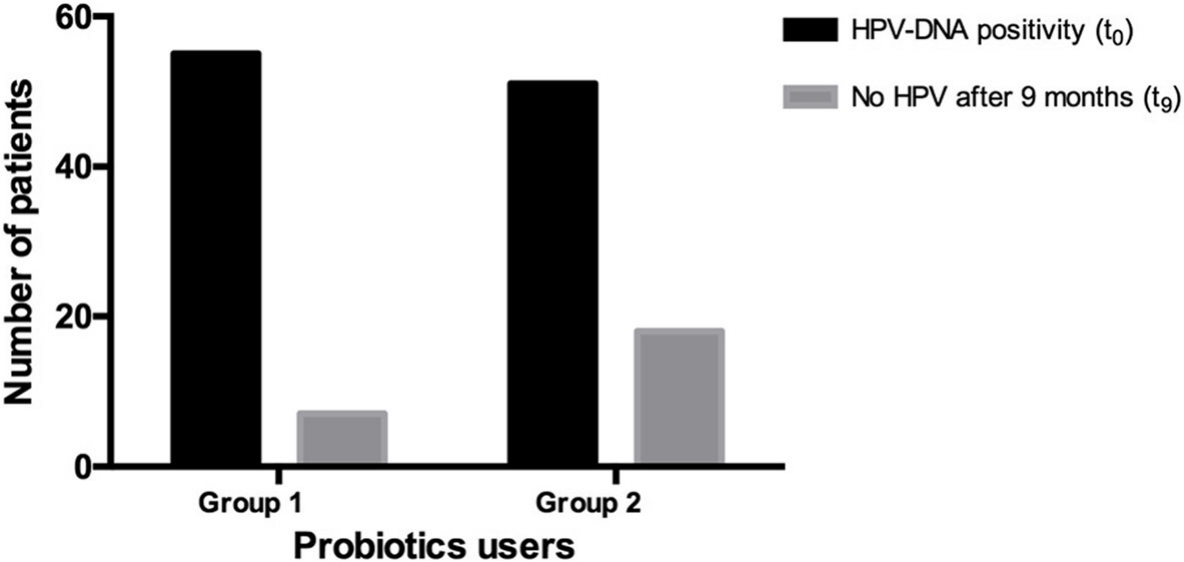
HPV clearance at the end of follow up period (t9) in the two groups (group 1 – short probiotics implementation group, *n* = 60; group 2 – long probiotics implementation group, *n* = 57). HPV clearance was significantly higher in long term probiotics users than in the other group (*p* = 0.044). t_0_: before treatment; t_9_: after 9 months follow up

At the end of the study period, the chance to solve HPV-related cytological anomalies was twice higher in *Lactobacillus rhamnosus* BMX 54 long-term vaginal users (79.4%, *n* = 31 vs 37.5%, *n* = 15; *p* = 0.041) as shown in Fig. 2. Moreover, a total HPV-clearance was shown in 11.6% (*n* = 7) of short treatment protocol patients compared to a percentage of 31.2% (*n* = 18) in long-term probiotic users (as negative HPV-DNA test) after 9 months since treatment starting (*p* = 0.044; Fig. 3).

Furthermore, long term probiotics administration determined also a reduction in vaginal infections recurrences as shown in Table 2.

**Table 2:** Follow up characteristics

	GROUP 1 (*n* = 60)				GROUP 2 (*n* = 57)			
FOLLOW UP (months)	t_0_	t_3_	t_6_	t_9_	t_0_	t_3_	t_6_	t_9_
Bacterial infections (n, %)	33 (55.0%)	1 (1.6%)	0 (0%)	2 (3.3%)	31 (54.4%)	1 (1.7%)	0 (0%)	0 (0%)
Yeast infections (n, %)	27 (45.0%)	3 (5.0%)	0 (0%)	7 (11.6%)	26 (45.6%)	1 (1.7%)	0 (0%)	1 (1.7%)
PAP smear abnormalities (n, %)	40 (66.6%)	28 (46.6%)	–	25 (41.6%)	39 (68.4%)	25 (43.8%)	–	8 (14.0%)
HPV DNA+ (n, %)	55 (91.7%)	–	–	48 (80.0%)	51 (89.4%)	–	–	33 (57.8%)
Pathologic colposcopy findings (HPV DNA negative) (n, %)	5 (8.3%)	2 (3.3%)	3 (5.0%)	3 (5.0%)	6 (10.5%)	3 (5.2%)	2 (3.5%)	0 (0%)

## Discussion

The vaginal microbiota plays a significant role in the female reproductive tract wellness maintenance. Although the human papilloma-virus is highly widespread, only a small number of women have recurrent HPV infection and subsequently develop precancerous lesions. An increased variety of vaginal microbiota, combined with a reduction of *Lactobacillus* spp., is linked with HPV infection acquisition and persistence and development of cervical pre-cancerous lesions and after years cancer [15–17]. Cervical cancer is the most common cancer among women in developing countries and the second most frequent female tumour worldwide [16]. Commonly, it progresses through a sequence of premalignant lesions known as cervical intraepithelial neoplasia (CIN) 1, 2 and 3 [9]. A normal cervical epithelial cell needs around 10–20 years to become malignant and just a few women with CIN lesions develop invasive cancer. HPV is a known risk factor for cervical cancer, but despite the high prevalence of HPV infection, CIN occurrence and evolution rates in untreated CIN lesions are quite low [17, 18]. As acknowledged from the literature, over 90% of HPV infections and infection-induced lesions are transient or recurrent and resolve spontaneously [19, 20]. Although HPV infection plays a key role in cervical cancer pathogenesis and develpoment, other environmental and host factors are involved in endorsing the process, as local microflora imbalance, immune response deficiency and consequently concomitant vaginal infections [21–24].

Physiologically, a healthy vaginal ecosystem is dominated by Lactobacilli and most of them had also a local immune system and inflammation response modulation [25, 26] and by controlling cell proliferation/apoptosis [27–29].

Hydrogen peroxide, lactate and bacteriocins made by Lactobacilli play an essential role in vaginal flora balance and eubiosis by inhibiting further pathogens’ colonization [13, 30]. Lactobacilli inhibit pathogen overgrowth in the whole urogenital tract, being a vital support in achieving a good reproductive and general health in women.

Importance of chronic inflammation in the development of pre-cancerous lesions has conquered increasing attention in the recent years and vaginal infections have been extensively debated in the literature as a risk factor for cervical dysplasia [31, 32].

Owing to a defined antitumor effect of probiotics, their fundamental role in vaginal ecosystem, their inhibitory effects on pathogens overgrowth and the relationship between vaginal infections and CIN, we assumed a possible relationship between vaginal Lactobacilli deficiency and HPV infections and consequently with HPV related cervical dysplasia.

*Lactobacillus rhamnosus* vaginal administration was chosen because it represents the exclusive *Lactobacillus* strain that has been recognized as able to colonize human vaginal microbiota once exogenously applied [26]. Thus, Lactobacilli BMX 54 – a specific selected *Lactobacillus* strain deposited to Pasteur Institute under Budapest Treaty –has shown clinical evidence of effectiveness when applied vaginally by restoring vaginal balanced ecosystem [33–36].

This study has some limitations the small sample size and the short follow up even if the statistically significant results obtained by using long-term probiotic vaginal administration are interesting and promising. In this study, cytological and viral endpoints were used to evaluate a potential effect of vaginal Lactobacilli long-term implementation in solving cervical abnormalities, through the establishment of the physiological vaginal balance (eubiosis). The consistent percentage of clearance of PAP-smear abnormalities obtained in vaginal Lactobacilli long term users group is interesting and encouraging and it supports our vaginal eubiosis versus pathobiosis theory.

## Conclusions

To our knowledge, this is the first study showing a control of HPV infection using probiotics. From our data, probiotics implementation for at least 6 months may be useful in re-establishing vaginal microflora and due to the recreation of vaginal balance, seems to benefit in controlling HPV infection.

## Abbreviations

ASCUS: Atypical Squamous Cells of Undetermined Significance
CFU: Colony-Forming Unit
CIN: Cervical Intraepithelial Neoplasia
HPV: Human Papilloma Virus
H-SIL: High Grade Intraepithelial Lesion
L-SIL: Low Grade Intraepithelial Lesion
PCR: Polymerase Chain Reaction
